# Hydrophobically Functionalized Poly(Acrylic Acid) Comprising the Ester-Type Labile Spacer: Synthesis and Self-Organization in Water

**DOI:** 10.3390/polym12051185

**Published:** 2020-05-22

**Authors:** Łukasz Lamch, Sylwia Ronka, Izabela Moszyńska, Piotr Warszyński, Kazimiera A. Wilk

**Affiliations:** 1 Department of Engineering and Technology of Chemical Processes, Faculty of Chemistry, Wrocław University of Science and Technology, Wybrzeże Wyspiańskiego 27, 50-370 Wrocław, Poland; izabela.moszynska@pwr.edu.pl; 2 Department of Engineering and Technology of Polymers, Faculty of Chemistry, Wrocław University of Science and Technology, Wybrzeże Wyspiańskiego 27, 50-370 Wrocław, Poland; sylwia.ronka@pwr.edu.pl; 3 Jerzy Haber Institute of Catalysis and Surface Chemistry, Polish Academy of Sciences, Niezapominajek 8, 30-239 Kraków, Poland; ncwarszy@cyf-kr.edu.pl

**Keywords:** hydrophobically functionalized polyelectrolytes, modified poly(acrylic acid), pH-sensitive structures, self-aggregation in aqueous solution, NMR diffusometry, molecular dynamics simulations

## Abstract

One of the most important properties of hydrophobically functionalized polyelectrolytes (HF-PEs) and their assemblies is their ability to encapsulate hydrophobic/amphiphilic agents and provide release on demand of the entrapped payload. The aim of the present work was to synthesize and study self-organization behavior in aqueous solution of hydrophobically functionalized poly(acrylic acid) (PAA) comprising the ester-type pH labile moiety with various degrees of hydrophobization and side-chain lengths in the absence and presence of appropriate mono- and polyvalent electrolytes (i.e., NaCl or CaCl_2_). The synthesis and purification of hydrophobically functionalized PAA were performed under mild conditions in order to avoid chemical degradation of the polymers. The modified polyelectrolytes self-assembly in aqueous systems was monitored using diffusion-ordered nuclear magnetic resonance (DOSY NMR). The performed studies, supported by the all-atoms molecular dynamics simulations, revealed a strong dependence of polyelectrolyte self-assembled state on concentration—specific concentration regions with the coexistence of both smaller and larger aggregates were observed (values of hydrodynamic diameter D_H_ around one nanometer and between two to six nanometers, respectively). Our investigations enabled us to gain crucial information about the self-assembly of the hydrophobically functionalized poly(acrylic acid) and opened the possibility of understanding and predicting its performance under various conditions.

## 1. Introduction

Due to their unique self-assembly properties, amphiphilic polymers constitute valuable specialty products, yielding nanocarriers for biomedical purposes, engineered surfaces as well as viscosity modifiers for various applications. The aggregate morphologies of non-charged copolymers are controlled by thermodynamic equilibrium: a balance between interfacial tension and chain stretching energies [1]. On the other hand, charged amphiphilic polymers, i.e., hydrophobically functionalized polyelectrolytes (HF-PEs), may be divided into two general groups: amphiphilic polyelectrolytes with ionizable groups, separated by hydrophobic units (like hexamethylene), comprising alternating hydrophilic/hydrophobic linear structures [2] (diblock copolymers with hydrophilic and hydrophobic blocks being a special case) and hydrophobically modified polyelectrolytes composed of polyelectrolyte (PE) backbone with hydrophobic side groups, such as shorter or longer alkyl chains, aromatic rings, etc. [3]. HF-PEs exhibit more complicated self-assembly behavior in comparison to their non-charged counterparts, mostly due to the balance between long-range electrostatic and short-range attraction interactions as well as hydration and counterion distribution. The most important feature of HF-PEs is their ability to self-assemble into a number of stimuli-responsive morphologies, which may undergo phase transitions or perform a release of solubilized/entrapped payload upon minor changes in temperature, pH, ionic strength, or by external physical stimuli (e.g., electric and magnetic fields) [4,5]. The above-mentioned specialty polymers may play a role as responsive aggregates for various applications, e.g., from drug and gene delivery to tissue engineering and battery electrolyte materials [1,6,7]. For functionalized polymers comprising PE backbone with hydrophobic side groups, the pH-responsivity is connected with protonation/deprotonation of weak or moderate electrolyte groups as well as hydrolysis of labile bonds between backbone and side chains. Generally, the reversible stimuli-responsive nature of HF-PEs in their aqueous solutions is associated with the moderation of hydrogen bonds, e.g., via the introduction of appropriate electrolyte (the influence of ionic strength on hydrogen ions activity) as well as pH or temperature changes.

Synthetic strategies for amphiphilic polyelectrolytes may include polymerization or step-by-step reactions involving monomers/building blocks of both hydrophilic and hydrophobic character as well as functionalization of an existing polymer, including their quaternization, esterification, amidation, etc., resulting in a polymer grafted with hydrophobic side groups. The first group of methods may be employed for the preparation of modified polyelectrolytes with a regular sequence of hydrophobic and hydrophilic units, mostly block and alternating ones. Approaches, relying on step-growth polymerization of different end groups or relative reactivities of monomers in copolymerization, followed by appropriate post-synthesis treatment (most often hydrolysis of end groups or anhydride bonds in the main chain), were applied in fabrication of poly(hexamethylene citramide) [8], and numerous copolymers of maleic acid with alkylvinylethers or 1-olefins, containing long alkyl fragments [9,10]. Furthermore, modification of existing polyelectrolytes or their precursors with hydrophobic molecules, involving alteration of reactive side groups, most often lead to polymers with randomly distributed hydrophobic groups. [3] The mentioned synthetic routes were used to prepare a variety of hydrophobically functionalized polymers, including poly(4-vinyl pyridine)—the first synthesized amphiphilic polyelectrolyte known as “polysoap” [11], chitosan [12], and poly(acrylic acid) [6,10], employing direct quaternization, reaction with fatty aldehydes, followed by reduction and amidation, respectively, to form stabile or labile bonds between side chains and the backbone. Hydrophobically functionalized statistical copolymers may be obtained when relative reactivities between monomers (e.g., dimethyl diallyl ammonium and methyl - n – alkyl diallyl ammonium salts) are similar to each other. Additionally, the regular or irregular structure of HF-PEs has a high impact on their aggregation properties. PEs with block structure, i.e., containing a hydrophilic part and a fragment composed exclusively of hydrophobic units, tend to form micelle-like aggregates of a few macromolecules. Moreover, HF-PEs with a random distribution of side chains can form intra- or inter-pseudo-micelles, depending on the content of lipophilic units [3]. An additional point of interest concerns the presence of an appropriate linking group between the fragments of extreme hydrophobicity—preferably a hydrolysable moiety like ester, secondary or tertiary amide, acetale, carbamate or urea—in order to attain sufficient biodegradability and reduced toxicity to higher organisms. The linker group may also contribute an added value to responsivities of HF-PEs to stimuli, due to its influence on weak interactions within the polyelectrolyte backbone [1,2,3,4,5]. The use of pH-sensitive polymers in drug delivery systems (DDSs) enables targeted payload release, which increases treatment efficiency as well as minimizing side effects. Great interest in the poly(acrylic acid) as a building material for DDSs is also linked with the possibilities of its easy modification. Therefore, in the last decade various researchers have focused their approaches on developing new drug-carrier systems based on poly(acrylic acid) (PAA) as well as poly(methacrylic acid) materials [7,10,13].

**Figure 1:**
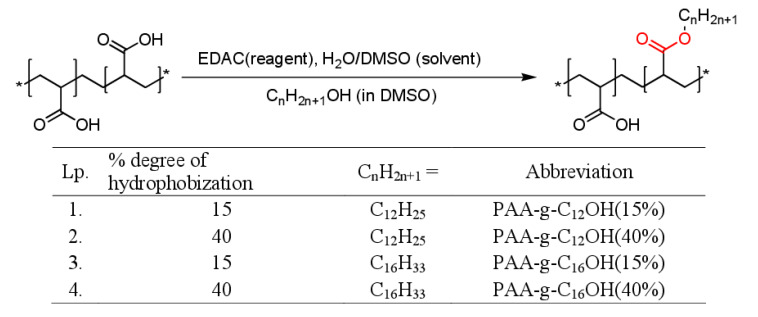
Chemical structure, abbreviations and synthetic route for hydrophobically functionalized poly(acrylic acids) (PAA) comprising the ester bond as a labile moiety. *N*-Ethyl-*N*’-(3-dimethylaminopropyl)carbodiimide hydrochloride (EDAC), dimethyl sulfoxide (DMSO).

As a continuation of our research on design, fabrication, and characterization of polymeric nanocarriers [13,14,15,16], our present contribution has aimed to investigate self-assembly behavior of newly synthesized hydrophobically functionalized poly(acrylic acid) (HF-PAA) with ester bonds linking alkyl side chains to polyelectrolyte backbone (see structures and abbreviations in Figure 1). Generally, it needs to be recalled that the aggregation of any HF-PEs can play a crucial role in prediction and moderation of their performance for multifunctional smart nanocarriers, emulgators, and viscosity modifiers toward numerous applications. Due to the presence of tiny aggregates, the adopted research methodology included high-resolution NMR techniques and the experimental work was supported by the results of the molecular dynamics simulations. We hypothesize that the self-assembly process of hydrophobically functionalized poly(acrylic acid) is very sensitive to subtle changes of pH and that it is possible to gain important information about formation of “local micelles“—characterized by the presence of internal hydrophobic nanodomains and outer, charged hydrophilic coronas. The presence of larger structures can be determined by indirect measurements of ionic strength influence upon aggregation behavior, i.e., upon addition of strong electrolyte. Combination of labile ester linking group between side chains and polyelectrolyte backbone and a weak (carboxylic acid) electrolyte - PAA (PAA-g-C_n_H_2n+1_OH; n = 12 and 16, set of derivatives with 15% and 40% percent degree of hydrophobization) may open new dual responsive characteristics of pH-sensitive polymer, connected with reversible protonation of acrylic acid moieties and irreversible hydrolysis of the linker groups.

## 2. Materials and Methods

### 2.1. Materials

Poly(acrylic acid) (PAA) (M_w_ = 100,000 Da, 35 wt % in H_2_O; data provided by supplier) was obtained from Sigma-Aldrich (Poznań, Poland). Coupling agents (*N*-Ethyl-*N*’-(3-dimethylaminopropyl)carbodiimide hydrochloride (EDAC; 98%) and 4-*N*,*N*’-dimethylaminopyridine (DMAP; 98%)) were purchased from Sigma-Aldrich (Poznań, Poland). Fatty alcohols (reagent grade, 98%)—dodecanol and hexadecanol—were obtained from Reachim (Moscow, Russia) and Fluka (St. Gallen, Switzerland), respectively. All other reagents and solvents were purchased from Avantor Performance Materials (Gliwice, Poland). All materials were used without further purification. All experiments and synthetic/purification procedures were performed using deionized water (Millipore).

### 2.2. Characterization

The functional groups were determined using a ThermoFisher Scientific Nicolet iS10 FT-IR spectrometer, equipped with VariGATR grazing angle accessory. Chemical structures were assessed by ^1^H NMR, recorded on a Bruker AMX-500 instrument. The chemical shifts in ^1^H NMR spectra are referenced to deuterium hydrogen oxide (semiheavy water, HDO) residual signal as an internal standard. The prepared grafted polyelectrolytes were dissolved in D_2_O before analysis. FT-IR and ^1^H NMR spectra of HF-PAAs are shown in Figures S1–S4 in the Electronic Supplementary Information (ESI).

### 2.3. Synthesis of the Hydrophobically Functionalized Poly(Acrylic Acid)

A 9.2% solution of poly(acrylic acid) (PAA) in distilled water was prepared. For each 1 g of polyelectrolyte (13.89 mmol of -COOH groups), an appropriate amount of *N*-Ethyl-*N*′-(3-dimethylaminopropyl)carbodiimide hydrochloride (2.50 mmol or 6.67 mmol for the desired degree of hydrophobization equal to 15% and 40%, respectively) was added and mixed to dissolve. Then dimethyl sulfoxide (DMSO) and a catalytic amount of 4-*N*,*N*’-dimethylaminopyridine (DMAP) were added. Afterwards, dodecanol or hexadecanol (2.08 mmol or 5.56 mmol, depending on the desired degree of hydrophobization, equal to 15% and 40%, respectively) dissolved in DMSO was added to the mixture, which was stirred at 50 °C for 48 h. The ratio of water to DMSO was between 8:3 and 4:5 (*v/v*). After completion of the reaction, the precipitated *N*-Ethyl-*N*′-(3-dimethylaminopropyl)urea was removed by filtration followed by solution dialysis in distilled water (4 × 4L, 4 days, MWCO 3500). Then the obtained solution was additionally filtered and freeze-dried.

- PAA-g-C_12_OH(15%): Yield: 75%; IR/cm^−1^: 3435 (O-H, stretching), 2939 (C-H, stretching), 1736 (C=O, stretching–ester), 1716 (C=O, stretching–carboxylic acid), 1030 (C-O, stretching); ^1^H NMR/ppm D_2_O: -CH_3_ (0.89–0.92, t), -CH(CO)CH_2_- (1.23–2.45, broad m), -OCH_2_CH_2_- (2.70–2.72, m), -OCH_2_- (2.91–3.06, m)
- PAA-g-C_12_OH(40%): Yield: 68%; IR/cm^−1^: 3450 (O-H, stretching), 2945 (C-H, stretching), 1736 (C=O, stretching–ester), 1719 (C=O, stretching–carboxylic acid), 1023 (C-O, stretching); ^1^H NMR/ppm D_2_O: -CH_3_ (0.98–1.01, t), -CH(CO)- (1.31–2.58, broad m), -OCH_2_CH_2_- (2.79–2.81, m), -OCH_2_- (3.02–3.14, m)
- PAA-g-C_16_OH(15%): Yield: 72%; IR/cm^−1^: 3438 (O-H, stretching), 2932 (C-H, stretching), 1740 (C=O, stretching–ester), 1718 (C=O, stretching–carboxylic acid), 1044 (C-O, stretching); ^1^H NMR/ppm D_2_O: -CH_3_ (0.94–0.97, t), -CH(CO)- (1.27–2.50, broad m), -OCH_2_CH_2_- (2.76–2.79, m), -OCH_2_- (2.96–3.10, m)
- PAA-g-C_16_OH(40%): Yield: 64%; IR/cm^−1^: 3442 (O-H, stretching), 2939 (C-H, stretching), 1734 (C=O, stretching–ester), 1717 (C=O, stretching–carboxylic acid), 1029 (C-O, stretching); ^1^H NMR/ppm D_2_O: -CH_3_ (0.96–0.99, t), -CH(CO)- (1.30–2.53, broad m), -OCH_2_CH_2_- (2.76–2.79, m), -OCH_2_- (2.99–3.12, m)

### 2.4. NMR Measurements

All NMR experiments were conducted on a Bruker AMX500 instrument in deuterated water at the stabilized temperature of 298 K. In ^1^H NMR spectra, chemical shifts were referenced to HDO signal (4.71 ppm) with a spectral resolution of at least 0.730 Hz (typically around 0.100–0.200 Hz). Samples were prepared by the direct dissolution of hydrophobically modified polyelectrolyte in D_2_O, followed by equilibration at room temperature for at least 1 h.

#### Diffusion Coefficients and Hydrodynamic Diameters Using Diffusion-Ordered Nuclear Magnetic R‘esonance (DOSY NMR)

DOSY NMR experiments were performed for hydrophobically functionalized PAA of different concentrations utilizing dstebpgp3s Bruker pulse program. The gradient amplitude γ and the maximum (initial) gradient strength G were constant and set at 4.258 × 10^8^ Hz/T and 45.74 T/m, respectively. The values of diffusion time Δ and gradient duration δ were chosen for each experiment individually in order to achieve the loss of integrated intensity values from 100% to 2%–7% during the experiment. Peak areas (integrated intensity for peaks with chemical shifts of around 1 ppm, attributed to methyl groups at the end of alkyl chain) for at least 14 increments of different gradient strength were used for each measurement. A mono-, bi-, and tricomponent functions:

(1) $$A=\sum_{i}A_{i}\exp\left(-D_{i}\left(\Delta -\frac{\delta }{3}\right){\left(\delta \;\gamma \;G_{i}\right)}^{2}\right)$$

were fitted to the data, where the total intensity (A) is a weighted sum of individual contributions (A_i_) and diffusion coefficients (D_i_) of differently diffusing populations.

Number weighted hydrodynamic radii were calculated using the Stokes-Einstein equation:

(2) $$R_{h}=\frac{kT}{6\;\pi \;\eta \;D}$$

where: Rh is the hydrodynamic radius, k is the Boltzmann constant (=1.38 × 10^-23^ m^2^kg/s^2^/K), T is the temperature in Kelvin (=298 K), η is the viscosity for D_2_O (1.09 mPas at 298 K), and D is the diffusion coefficient determined above. The impact of NaCl and CaCl_2_ on the self-organization of hydrophobically functionalized poly(acrylic acid) was studied for PAA-g-C_16_OH(40%) at a concentration of 45 mg/mL. Samples containing inorganic salts were prepared by mixing appropriate amounts of PAA-g-C_16_OH(40%) and NaCl or CaCl_2_ followed by equilibration (at least 5 min before DOSY experiment).

### 2.5. Molecular Dynamics Modeling

In our previous works, we used molecular dynamics simulation to study the dependence of the conformation of the polyelectrolyte chain and its effective charge on the electrolyte concentration and the degree of ionization for poly(acrylic acid) [17]. Katiar and Jha used atomistic molecular dynamics modeling to investigate the phase behavior of aqueous poly(acrylic acid) solutions [18]. Sulatha and Natarajan used single chain molecular dynamics simulation to study the effect of PAA charging on adsorption on dodecyltrimethylammonium chloride micelle in water [19]. Liu et al. [1] used coarse-grain model molecular dynamics to simulate the self-assembly behavior of diblock copolymers consisting of one hydrophobic and one ionizable polyelectrolyte (PE) block in the presence of monovalent and multivalent counterions. Emamyari and Fazli investigated conformations of a model comb polyelectrolyte with a hydrophobic backbone and charged hydrophilic side chains [20].

To study the formation of intra-molecular aggregates and the formation of hydrophobic domains, we performed the all-atoms molecular dynamics simulations of the hydrophobically modified poly(acrylic acid), PAA-g-C_12_OH(15%). Due to the computing time limitation, we simulated the copolymer with a molecular weight of 7.8 kD, consisting of 80 monomers. We used YASARA molecular modeling software [21] with AMBER14 force field [22]. To elucidate the role of polyelectrolyte backbone charging degree we manually assigned the protonation state to obtain 100% charged units (i.e., the total charge of the molecule -68e), 44%, 28%, 18%, and 0% (i.e., uncharged chain corresponding to full protonated state). Atomic fractional charges and bond orders were assigned using the *AutoSMILES* procedure contained in the YASARA Structure package [21]. At the beginning of the simulation, the copolymer in the initial configuration, as illustrated in Figure S5 in the ESI, was placed in the simulation box, 15 nm × 15 nm. The cell was filled with water molecules (TIP3P, density 1 g/cm^3^) and Na^+^ and Cl^-^ ions to obtain electroneutrality and 0.015 M NaCl salt concentration. The electrostatic interactions were calculated using the particle mesh Ewald approach [23] while the 1.0 nm cut-off was used for the van der Waals interactions. Simulations ran at 298 K.

## 3. Results and Discussion

### 3.1. Design, Synthesis, and Characterization of the Hydrophobically Functionalized Poly(Acrylic Acid)

The HF-PAA product was designed as a multifunctional material comprising both weak electrolyte moieties (carboxylic acid) and alkyl side chains attached to the polymeric backbone via the ester linkage. The abovementioned structural features may open possibilities to achieve the stimuli-responsivity behavior for even slight changes in solution composition followed by enhanced chemo- and biodegradability of the material after its usage. The mentioned properties are highly desired for any materials designed toward biomedical purposes. The synthetic routes for hydrophobically functionalized poly(acrylic acid) are presented in Figure 1. Accordingly, the designed hydrophobized PAAs were synthesized in mild conditions (Steglich esterification in water/DMSO mixtures, utilizing *N*-Ethyl-*N*′-(3-dimethylaminopropyl)carbodiimide hydrochloride as coupling agent). The starting materials and synthetic routes were chosen in order to gain specific application-related features: high electrolyte tolerance combined with pH dependence moderated by ionic strength as well as excellent dispersibility in aqueous systems (up to concentration exceeding 10% *w*/*w*—see Table 1). The intended modalities were obtained by a combination of weak electrolyte groups in poly(acrylic acid), known for its excellent solubility even in concentrated electrolyte solutions, with labile ester bonds for coupling of hydrophobic moieties. Moreover, the addition of electrolyte to poly(acrylic acid) solution has a proven influence on bulk (strong viscosity dependence) and surface (moderation of surface tension) properties and opens unique application features for its hydrophobically functionalized derivatives [24].

The chemical structures of the synthesized hydrophobically functionalized poly(acrylic acid) were confirmed utilizing ^1^H NMR and FT-IR spectra (see Figures S1–S4 in Electronic Supplementary Material for FT-IR and ^1^H NMR spectra). FT-IR spectra showed the presence of characteristic bands, attributed to C-H, C-O, O-H, and C=O bonds, confirming their occurrence in the synthesized macromolecules. The presence of both ester and carboxylic acid groups is indicated by the stretching band of carbonyl at around 1735–1740 cm^−1^ and around 1715–1720 cm^−1^, respectively. In the obtained ^1^H NMR spectra (for PAA modified with dodecanol—PAA-g-C_12_OH(15%) and PAA-g-C_12_OH(40%)—or hexadecanol—PAA-g-C_16_OH(15%) and PAA-g-C_16_OH(40%)—at the concentration equal to 10 mg/mL), we can see sharp signals attributed to dodecyl or hexadecyl alkyl fragments with significant triplet for chemical shift below 1 ppm (methyl group at the end of chain). Moreover, broad signals attributed to aliphatic CH and CH_2_ moieties in the polyelectrolyte backbone are also present in the range between 1.3 and 2.3 ppm. The most important feature of the synthesized hydrophobically functionalized PAA is a significant dependence of the signals chemical shifts on hydrophobization degree (15% or 40%). Generally, the values of chemical shifts were significantly higher (difference up to around 0.1 ppm) for products with a hydrophobization degree of 40% in comparison to PAA modified in 15%. No significant dependence on concentration, length of alkyl chain, or aggregation was observed. The mentioned effect was more visible for PAA modified with dodecanol and is probably related to the number of ionizable groups, influencing ionic strength, different for 15% and 40% hydrophobization degree.

### 3.2. NMR Diffusometry Studies upon Self-Organization of Hydrophobically Functionalized Poly(Acrylic Acid)

Hydrophobically functionalized polyelectrolytes, i.e., polyelectrolytes with covalently bound hydrophobic side groups, most often aromatic or long aliphatic chain, may be divided into two general groups: hydrophobically modified water-soluble polymers, so-called associating polymers, and polysoaps. The first group comprises a hydrophilic backbone with a small number (typically up to 2%) of strongly hydrophobic side chains with an increased tendency to form intermolecular aggregates, which may result in strong viscosity enhancements. Polysoaps, the second group, also have a polyelectrolyte backbone and hydrophobic side groups, but the content of the hydrophobic fragments may be even more than 10%. In aqueous solutions, they can produce local micelles (intramolecular pseudo-micelles) or intermolecular aggregates, depending on concentration, the ionizable groups’ character and overall increment of hydrophobic groups in the whole macromolecule (i.e., degree of hydrophobization) [4]. It is considered that polysoaps with flexible polyelectrolyte backbone and the appropriate distance between neighboring side chains are prone to form local micelles with mean hydrodynamic diameters comparable to low molecular weight surfactants micelles (generally, less than about 3 nm) [25].

Analyses of ^1^H NMR spectra constitute a very powerful tool toward colloidal systems, especially in the field of solubilization in self-assembled structures [13,15]. On the other hand, chemical shifts are significantly more dependent on pH in comparison to other factors (e.g., screening effects, proton-proton interactions through space or mobility) [15]. That is why the mentioned techniques are limited to the systems with very low influence of the pH values—even a slight addition of acidic/basic compound may drastically change chemical shift. HF-PAAs, like any weak acids, significantly influence overall pH of solution as well as, due to its polymeric structure, may form nano- and microdomains with differing local acidity. Values of chemical shifts in the given solutions of HF-PAAs are accurate only to compare different systems rather than study the influence of aggregation on signals in the ^1^H NMR spectra. Taking into consideration the abovementioned limitations of proton shifts analysis we have chosen DOSY NMR technique for our investigations of the HF-PAAs self-assembly behavior.

Our studies on the aggregation of hydrophobically modified poly(acrylic acid) concern two products with the utmost content and length of alkyl side chains: PAA-g-C_12_OH(15%) and PAA-g-C_16_OH(40%). These compounds were carefully studied exploiting diffusion-ordered nuclear magnetic resonance (DOSY NMR)—one of the most widespread NMR approaches in the field of colloids chemistry and physics—in order to find hydrodynamic diameters of their aggregates as well as to determine the qualitative and quantitative influence of electrolyte addition on the mentioned self-assembly processes.

**Figure 2:**
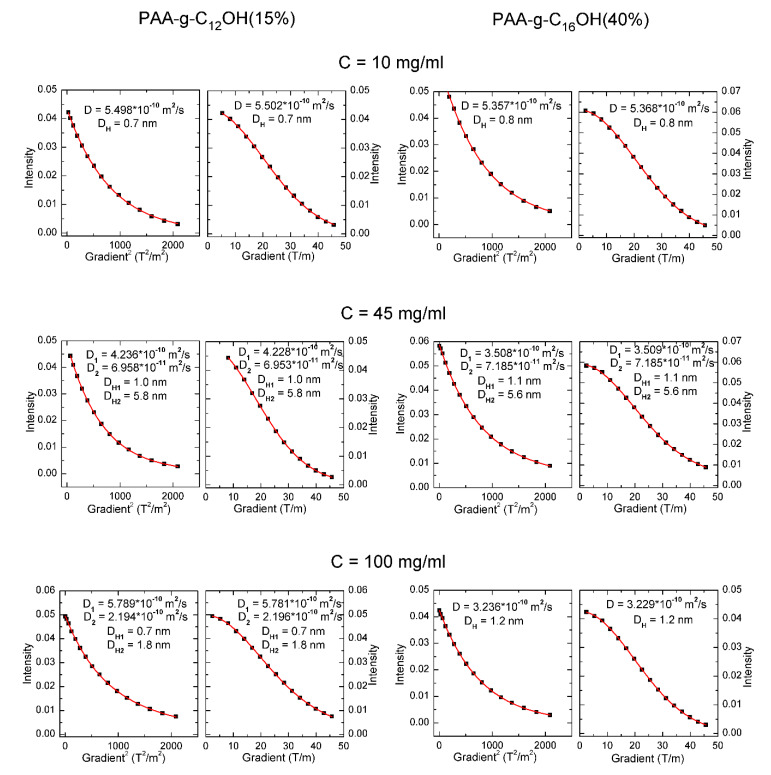
Diffusion coefficients (D) and hydrodynamic diameters (D_H_) determined by fitting to appropriate models: G (intensity versus linear gradient) or G^2^ (intensity versus square gradient).

Diffusion ordered nuclear magnetic resonance (DOSY NMR) constitutes a very useful technique for complex analysis of self-assembly in disperse systems (e.g., surfactants [26], block copolymers [27,28]), as well as other dynamic systems (e.g., micro- and nanoemulsions [29] and aggregating complex ions [30]). The mentioned approach has been employed in the determination of critical micelle/critical aggregation concentrations [31] and hydrodynamic diameters of unimers and macromolecular complexes via the Stokes-Einstein equation [27]. The DOSY NMR studies were performed for PAA-g-C_12_OH(15%) and PAA-g-C_16_OH(40%) (products with extreme overall hydrophobicity) at three concentrations: 10 mg/mL, 45 mg/mL, and 100 mg/mL. The integrated intensities of methyl protons at the end of hydrophobic alkyl chains (δ_H_ ≈ 0.9–1.0 ppm) were plotted versus linear gradient (model G^1^ in Table 1) and square gradient (model G^2^ in Table 1) and fitted to appropriate mono- and bicomponent functions, representing uni- and bimodal distributions, respectively. The best possible fittings for each system (good agreement between G^1^ and G^2^ models with the lowest possible values of adjusted R^2^ coefficient) are marked with bold font. The fitted functions and data points (for the marked functions from Table 1) are presented in Figure 2.

We also tried to fit the obtained data to tricomponent functions but an overparametrization was observed (mutual dependence between parameters, resulting in worse fitting in comparison to an equation with a lower number of parameters)—see Table S1 in ESI. Bimodal distribution was found to be the most appropriate for PAA-g-C_12_OH(15%) at concentrations equal to 45 mg/mL and 100 mg/mL as well as PAA-g-C_16_OH(40%) at concentration of 45 mg/mL. For the mentioned samples, there were found two types of aggregates with diffusion coefficients equal to about 3.5 × 10^−10^–4.5 × 10^−10^ m^2^/s and around 7 × 10^−11^ m^2^/s, representing, most probably, intramolecular pseudo-micelles and higher aggregates, respectively. Unimodal distribution was optimal for PAA-g-C_12_OH(15%) at concentration 10 mg/mL as well as PAA-g-C_16_OH(40%) at concentrations equal to 10 mg/mL and 100 mg/mL—for the mentioned samples only aggregates with diffusion coefficients equal to about 3.0 × 10^−10^–5.5 × 10^−10^ m^2^/s were present. Diffusion coefficients of PAA-g-C_12_OH(15%) and PAA-g-C_16_OH(40%) solutions, regardless their concentration, were around 10^−11^–10^−10^ m^2^/s, so the calculated from Stokes-Einstein equation hydrodynamic diameters were equal to around 1 nm (smaller aggregates—possibly intramolecular pseudo-micelles) and 5–6 nm (larger aggregates).

Thus, our studies show the strong dependence of aggregate diffusibility (and values of D_H_) on overall hydrophobicity in solution. In diluted (concentration–10 mg/mL) solutions of PAA-g-C_12_OH(15%) and PAA-g-C_16_OH(40%) only small aggregates are present, with values of D_H_ around 0.7–1.3 nm. For solutions with higher overall hydrophobicity (higher concentration and/or length and number of hydrophobic units per polyelectrolyte backbone), bimodal systems are present with smaller (0.7–1.3 nm) and larger (5.5–5.8 nm) aggregates. Contrary to intuition, the solution with the highest overall hydrophobicity (PAA-g-C_16_OH(40%) at a concentration of 100 mg/mL) contained only small aggregates with D_H_ equal to approximately 1.2 nm. These observations may result from the balance of hydrophobic and electrostatic forces in the mentioned solutions. Generally, electrostatic forces for diluted solutions of polyelectrolytes play a negligible role in the formation of aggregates—the mentioned process is mainly driven by hydrophobic forces, thus smaller, intramolecular aggregates are preferred in the system due to low probability of collision between two or more macromolecules [4]. Aqueous solutions of HF-PEs may tend to form larger, intermolecular aggregates above a certain concentration (generally around 0.1%–1.0%)—in this region, bimodal systems are observed. For semi-diluted solutions of HF-PEs with the highest overall hydrophobicity combination of electrostatic forces, hydrophobic interactions and steric effects stimulate generation of larger, mostly intermolecular, aggregates. Thus only smaller intermolecular pseudo-micelles may be present in the solution [3]—the mentioned effect is observed for PAA-g-C_16_OH(40%) at a concentration of 100 mg/mL. Our studies reveal that overall hydrophobicity and concentration of HF-PAA play crucial roles in their self-assembly behavior. Generally, there was observed optimal concentration/overall hydrophobicity range for formation of aggregates with higher values of hydrodynamic diameter. For diluted solutions, aggregation is driven mostly by the hydrophobicity of side alkyl chains so micelle-like aggregates are preferred. For more concentrated solutions, both hydrophobic and electrostatic interactions may rule the self-organization of HF-PEs.

**Figure 3:**
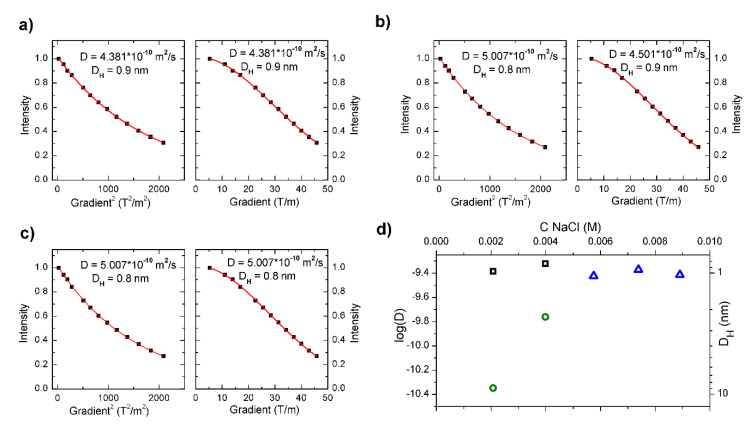
Effect of NaCl and CaCl_2_ on self-organization of PAA-g-C_16_OH(40%) (c = 45 mg/mL samples containing 0.05 M (**a**) and 0.15 M NaCl (**b**) as well as 0.05 M CaCl_2_ (**c**), along with calculated diffusion coefficients (log(D)) and hydrodynamic diameters (D_H_) plotted versus concentration of NaCl (**d**). Intramolecular pseudo-micelles (smaller fraction of aggregates) are denoted by black squares (for bimodal distribution) and blue triangles (for unimodal distribution), while larger aggregates by green circles.

In order to study the impact of NaCl and CaCl_2_ on the self-organization of PAA-g-C_16_OH(40%) (c = 45 mg/mL) samples containing 0.05 M and 0.15 M NaCl as well as 0.05 M CaCl_2_ were prepared (see Figure 3a–c). All the studied “survey samples” exhibited the best fitting to monoexponentional functions (unimodal distribution), attributed to (most probably) intramolecular pseudo-micelles with diffusion coefficients around 4.5 × 10^−10^ m^2^/s, corresponding to hydrodynamic diameters of ca. 0.9 nm (see Figure 3a–c). On the other hand, PAA-g-C_16_OH(40%) at a concentration of 45 mg/mL, without the addition of any salt, showed bimodal distribution (diffusion coefficients 3.5 × 10^−10^ m^2^/s and 7.2 × 10^−11^ m^2^/s with corresponding hydrodynamic diameters 1.1 nm and 5.6 nm, respectively). Studies of the mentioned “survey samples” enabled us to formulate a hypothesis that, above the certain (“critical”) value of salt concentration, the formation of higher aggregates (i.e., with hydrodynamic diameters exceeding ca. 1.3 nm) is strongly unfavorable. The process of PAA-g-C_16_OH(40%) self-organization in aqueous solution with increasing salt concentration was studied by means of DOSY NMR in order to test the hypothesis. For the mentioned experiment NaCl (simple 1:1-type strong electrolyte) was chosen as a model salt. The obtained data were fitted to appropriate models (mono- and biexponential) and the most relevant diffusion coefficients (decimal logarithm of values in m^2^/s and corresponding values of hydrodynamic diameters) were plotted versus increasing salt concentration (see Figure 3d). The obtained results revealed that the mean diameter of intramolecular pseudo-micelles (see Figure 3d, black squares in for bimodal distribution, blue triangles for unimodal distribution) practically does not change with increasing NaCl concentration, corresponding to their stability (the mentioned self-assembled structures are present at all concentrations of PAA-g-C_12_OH(15%) and PAA-g-C_16_OH(40%), sometimes coexisting with larger aggregates). Mean hydrodynamic diameters of larger aggregates (green circles in Figure 3d) significantly decrease with an increase of NaCl concentration from around 8.9 nm (D = 4.5 × 10^−11^ m^2^/s) in 0.002 M NaCl to 2.3 nm (D = 1.7 × 10^−10^ m^2^/s) in 0.004 M NaCl. For higher NaCl concentrations (above or equal to 0.006 M) larger aggregates are not present in the analyzed solutions. Decrease of poly(acrylic acid) self-assembled structures diameters with an increase of NaCl concentration was previously observed by Ishimuro [24] and attributed to a lower number of molecular coils as well as an amplified surface activity for higher values of ionic strengths. According to hydrophobically functionalized PAA-g-C_16_OH(40%), this effect may arise from the promoted formation of densely packed aggregates (ruled by lower values of electrostatic repulsive forces) with increasing salinity followed by adsorption of HF-PE at the interfaces. The similar effects, i.e., increased surface activity for higher values of ionic strength, is observed for other amphiphiles like low molecular weight surfactants [25].

Generally, DOSY NMR studies show that self-assembled structures of PAA-g-C_12_OH(15%) and PAA-g-C_16_OH(40%) are strongly dependent on the concentration of hydrophobically functionalized polyelectrolyte and exhibit a specific region with the presence of nanostructures with larger values of hydrodynamic diameter.

### 3.3. Molecular Dynamics Simulation of Self-Assembly of a Single Hydrophobically Functionalized Poly(Acrylic Acid)

**Figure 4:**
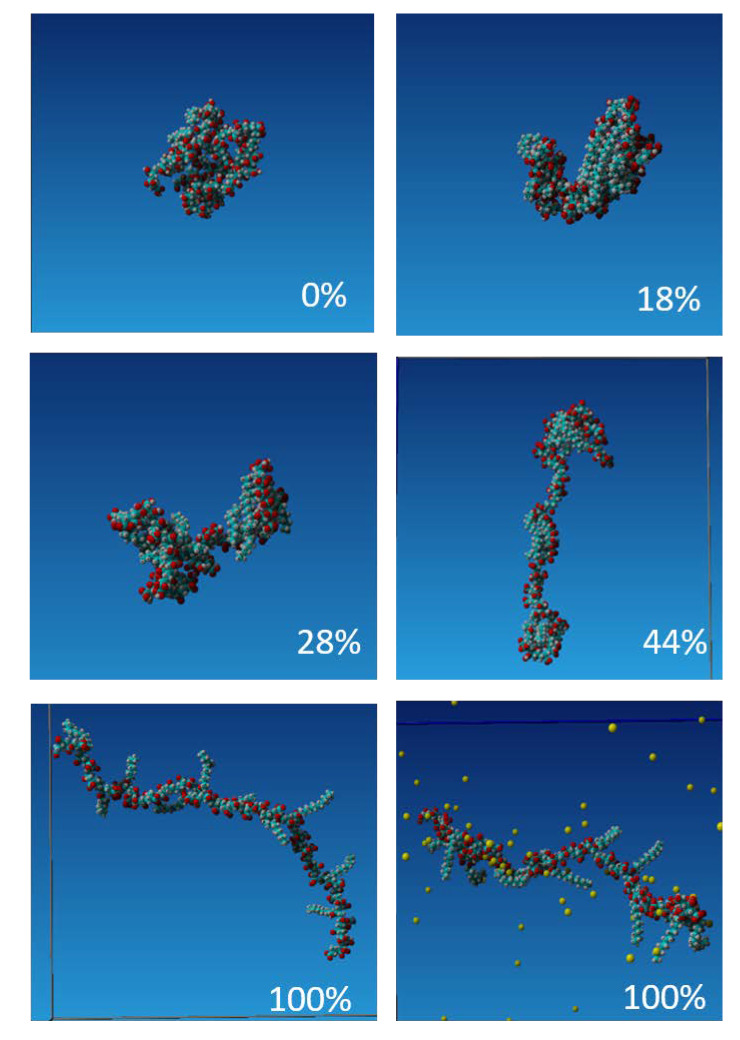
Snapshots obtained after 25 ns molecular dynamics simulations of PAA-g-C_12_OH(15%) with various degrees of charging (marked in the Figure). The condensation of Na^+^ counterions at 100% charged polymer is additionally shown. Legend: oxygen atoms are denoted in red, carbon atoms—blue, hydrogen atoms—grey and Na^+^ ions—yellow.

We performed the molecular dynamics simulation of the evolution of conformation of single poly(acrylic acid) polymer chain hydrophobically functionalized by 15% grafting of dodecyl tails, PAA-g-C_12_OH(15%). The chain consisted of 80 PAA monomers and the copolymer molecular weight 7.8 kD was c.a. 10 times lower than used in experiments. PAA-g-C_12_OH(15%) was chosen for molecular modeling studies due to its least complicated structure due to shorter hydrophobic chains, which decrease the demand for the computing time. However, the obtained results may be applied in order to explain the behavior of PAA-g-C_16_OH(40%), especially if the effect of the polymer charge is concerned. To investigate the formation of pseudo-micelles structures we ran the simulation for the five degrees of PAA charging, i.e., the carboxylic group deprotonation, 100% charged units (i.e., for fully deprotonated state), 44%, 28%, 18%, and 0% (i.e., uncharged chain corresponding to full protonated state). The representative snapshots obtained after 1 ns, 5 ns, 10 ns, and 25 ns after the start of the simulation with the identical initial conformation of the molecule (Figure S5) are illustrated in Figure S6. Water molecules and NaCl ions are not shown for the sake of clarity. One can notice that after 5 ns the grafted hydrophobic chains are collapsed onto the PAA backbone and the pseudo-micellar structures consisting of 2–3 hydrophobic moieties start to form. The exception is a fully charged chain; here the dodecyl chains are exposed to the aqueous environment despite their hydrophobicity. Figure 4 presents the details of the molecular conformation of PAA-g-C_12_OH(15%) obtained after 25 ns of simulations and illustrates the differences of the conformation depending on its total charge. For the uncharged chain, the initial pseudo-micelle developed into the blob that grew with time and the copolymer tended to assume globular conformation. For weakly charged polymer, 18% and 28% deprotonation, the molecule assumes a necklace or dumbbell conformation. The hydrophobic domains consisting of 2–3 dodecyl chains of the size c.a. 1 nm were developed while larger objects (blobs) consisting of several parallel oriented chains, to maximize the hydrophobic effect, may appear at the chain ends. The electrostatic interactions govern the size of these structures and the distance between them. For the moderately charged polymer, only small pseudo-micellar objects are present, formed by 2–3 dodecyl chains collapsed onto the PAA backbone. These structures are absent for fully charged (deprotonated) copolymer and the dodecyl chains are stretched into the aqueous environment. Strong hydration and presence of condensed counterions prevent the DD chains from collapsing.

The results of molecular dynamics simulations allowed proposing the interpretation of the outcome of DOSY NMR experiments. Since in our experimental conditions, the hydrophobically modified PAA was either moderately or weakly charged, we observed coexistence of interchain pseudo-micellar aggregates of c.a. 1 nm and larger ones predominantly developed at the ends of polymer chains. The size of these aggregates grew with the length of hydrophobic chains and their number increased with polyelectrolyte concentration. For the highest concentration of 100 mg/mL (PAA-g-C_16_OH(40%)), the formation of large aggregates was not observed that indicated that the interchain electrostatic interactions were strong enough to stabilize the polymer solution. On the other hand, for that concentration, the solution was in the semi-dilute range and the steric effects hampered the formation of end-chain blobs of the size of a few nanometers. The addition of salt screens for electrostatic interactions and simultaneously, for highly and moderately charged chains, increased the counterion condensation [32]. Spacing between charges of carboxylic groups of the PAA chain is 0.27 nm, while the Bjerrum length in water is 0.7 nm, so even at a 50% charged chain, the counterion condensation should be observed. As it was shown in the simulation counterion condensation together with strong hydration prevent the collapse of the hydrocarbon chains and formation of hydrophobic domains. On the other hand, addition of salt may impose osmotic stress on the large end-chain blobs that causes their destabilization. That was observed in DOSY NMR experiments after NaCl addition above 5 mM, as shown in Figure 3d. Further addition of salt above 0.15 M NaCl caused the system destabilization and phase separation (formation of polymer rich and polymer poor phases).

## 4. Conclusions

We studied self-assembly of the hydrophobically functionalized poly(acrylic acid) (HF-PAA)—a unique class of amphiphilic polyelectrolytes, combining labile (ester) linking group between side chains and polyelectrolyte backbone and weak (carboxylic acid) electrolyte. The mentioned strategy makes it possible to obtain dual responsive characteristics of pH-sensitive polymer: reversible protonation of acrylic acid moieties and irreversible hydrolysis of linker groups. The designed and obtained HF-PAAs exhibited very good aqueous solubility, despite the alkyl chain length (dodecyl or hexadecyl) and degree of hydrophobization (15% or 40% substitution of carboxylic acid groups), with visible viscosity enhancement. The chemical structures of the synthesized compounds were confirmed by ^1^H NMR and FT-IR spectroscopies. In order to assess information about very small (around one nanometer) self-assembled nanostructures our studies comprised diffusion ordered NMR and molecular dynamics modeling, performed for poly(acrylic acid) modified with 15% substitution with dodecanol (PAA-g-C_12_OH(15%)) and 40% substitution with hexadecanol (PAA-g-C_16_OH(40%)). Our results clearly indicate that pseudo-micelles with D_H_ of around one nanometer are present for both PAA-g-C_12_OH(15%) and PAA-g-C_16_OH(40%) at all studied concentrations in the range 10–100 mg/mL, sometimes coexisting with larger aggregates. The mean diameters of pseudo-micelles (around one nanometer) are in good agreement with approximate molecular lengths of alkyl chains comprising around 10–14 methylene units. On the other hand, these aggregates seem to be the most stable conformation of polyelectrolyte chains in aqueous systems and constitute building units of higher aggregates, especially with D_H_ of around six nanometers. Moreover, self-assembled structures of PAA-g-C_12_OH(15%) and PAA-g-C_16_OH(40%) are strongly dependent on the concentration—presence of specific concentration regions with the coexistence of both smaller and larger aggregates. Molecular dynamics simulations confirmed the formation of pseudo-micellar aggregates of the one nanometer size consisted of two to three hydrophobic chains collapsed onto the PAA backbone, except for highly charged polymers where the collapse is prevented by PAA hydration and presence of condensed counterions. The addition of salt increases the degree of condensation. Moreover, the presence of additional salt may induce osmotic stress on the larger aggregates and destabilize them. The significance of our investigations upon that unique hydrophobically functionalized polyelectrolyte opened the possibility of understanding its further performance properties, especially toward sophisticated applications in pH-responsive structures for drug delivery as well as viscosity modifiers.

## Figures and Tables

**Table 1:** Determination of diffusion coefficients (D) and hydrodynamic diameters (D_H_) by fitting data to appropriate models: G^1^ (intensity versus linear gradient) or G^2^ (intensity versus square gradient).

C [mg/mL]	Model (G^1^ or G^2^)	PAA-g-C_12_OH(15%)			PAA-g-C_16_OH(40%)		
		D [m^2^/s]	D_H_ [nm]	R^2^	D [m^2^/s]	D_H_ [nm]	R^2^
10	G^1^ (1 coefficient)	5.502 × 10^−10^	0.7	0.99977	5.368 × 10^−10^	0.7	0.99990
	G^1^ (2 coefficients)	6.352 × 10^−10^	0.6	0.99976	5.815 × 10^−10^	0.7	0.99989
		6.218 × 10^−10^	0.6		5.949 × 10^−10^	0.7	
	G^2^ (1 coefficient)	5.498 × 10^−10^	0.7	0.99977	5.357 × 10^−10^	0.8	0.99990
	G^2^ (2 coefficients)	5.498 × 10^−10^	0.7	0.99973	5.356 × 10^−10^	0.8	0.99989
		5.498 × 10^−10^	0.7		5.356 × 10^−10^	0.8	
45	G^1^ (1 coefficient)	4.007 × 10^−10^	1.0	0.99976	2.791 × 10^−10^	1.4	0.99645
	G^1^ (2 coefficients)	4.236 × 10^−10^	1.0	0.99996	3.508 × 10^−10^	1.1	0.99986
		6.958 × 10^−11^	5.8		7.185 × 10^−11^	5.6	
	G^2^ (1 coefficient)	4.016 × 10^−10^	1.0	0.99976	2.580 × 10^−10^	1.6	0.99645
	G^2^ (2 coefficients)	4.228 × 10^−10^	0.9	0.99996	3.509 × 10^−10^	1.2	0.99986
		6.953 × 10^−11^	5.8		7.185 × 10^−11^	5.9	
100	G^1^ (1 coefficient)	3.635 × 10^−10^	1.1	0.99761	3.236 × 10^−10^	1.2	0.99994
	G^1^ (2 coefficients)	5.781 × 10^−10^	0.7	0.99986	3.236 × 10^−10^	1.2	0.99993
		2.196 × 10^−10^	1.8		3.326 × 10^−11^	1.2	
	G^2^ (1 coefficient)	3.610 × 10^−10^	1.1	0.99745	3.238 × 10^−10^	1.2	0.99994
	G^2^ (2 coefficients)	5.789 × 10^−10^	0.7	0.99986	3.238 × 10^−10^	1.2	0.99993
		2.194 × 10^−10^	1.8		3.238 × 10^−10^	1.2	

## Supplementary Materials

The following are available online at https://www.mdpi.com/2073-4360/12/5/1185/s1, Figure S1: FT-IR spectrum of the synthesized hydrophobically functionalized poly(acrylic acid) in comparison to poly(acrylic acid), Figure S2: FT-IR spectrum of the synthesized hydrophobically functionalized poly(acrylic acid) with marked signals attributed to the main bonds, Figure S3: ^1^H NMR spectra of the synthesized hydrophobically functionalized poly(acrylic acid) (0–8 ppm range), Figure S4: ^1^H NMR spectra of the synthesized hydrophobically functionalized poly(acrylic acid) (0.5–3.5 ppm range), Figure S5: Initial conformation of hydrophobically modified PAA molecule used for the molecular dynamics simulations, Figure S6 Snapshots from the molecular dynamics simulations of PAA-g-C_12_OH(15%) with various degree of charging (marked in the Figure) taken after 1ns, 5 ns, 10 ns and 25 ns of the simulation run, Table S1: Determination of diffusion coefficients (D) and hydrodynamic diameters (D_H_) by fitting data to appropriate triexponentional models: G^1^ (intensity versus linear gradient) or G^2^ (intensity versus square gradient).
