# Supplementary material for: Hydrophobically Functionalized Poly(Acrylic Acid) Comprising the Ester-Type Labile Spacer: Synthesis and Self-Organization in Water
Source: Polymers (Basel). 2020 May 22;12(5):1185. doi: 10.3390/polym12051185 (PMC7285226; doi:10.3390/polym12051185)
Supplement: Supplementary file 1 [file polymers-12-01185-s001.pdf]

ELECTRONIC SUPPLEMENTARY MATERIAL

Hydrophobically functionalized poly(acrylic acid) comprising the ester-type labile spacer: Synthesis and self-organization in water

Łukasz Lamch[*a], Sylwia Ronka[b], Izabela Moszyńska[a], Piotr Warszyński[c], Kazimiera A. Wilk[*a]

[a)] Department of Engineering and Technology of Chemical Processes, Faculty of Chemistry, Wrocław University of Science and Technology, Wybrzeże Wyspiańskiego 27, 50-370 Wrocław, Poland

[b)] Department of Engineering and Technology of Polymers, Faculty of Chemistry, Wrocław University of Science and Technology, Wybrzeże Wyspiańskiego 27, 50-370 Wrocław, Poland

[c)] Jerzy Haber Institute of Catalysis and Surface Chemistry, Polish Academy of Sciences, Niezapominajek 8, 30-239 Kraków, Poland

* To whom correspondence should be addressed

phone: +48 71 3202828, fax: +48 71 3203678

email: lukasz.lamch@pwr.edu.pl (Łukasz Lamch), kazimiera.wilk@pwr.edu.pl (Kazimiera A. Wilk)

1. FT-IR, etc and ^{1}H NMR spectra

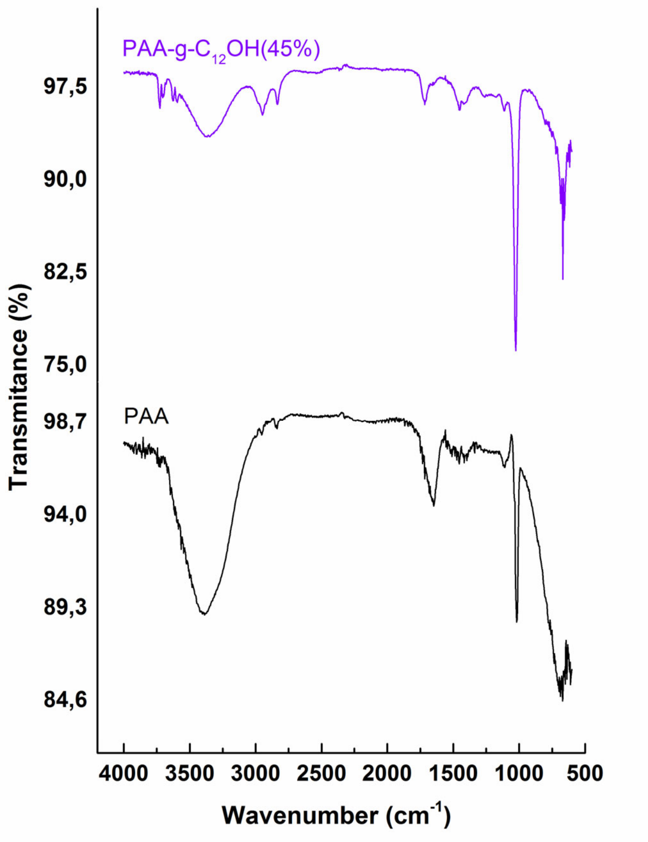

Figure 1S. FT-IR spectrum of the synthesized hydrophobically functionalized poly(acrylic acid) in comparison to poly(acrylic acid).

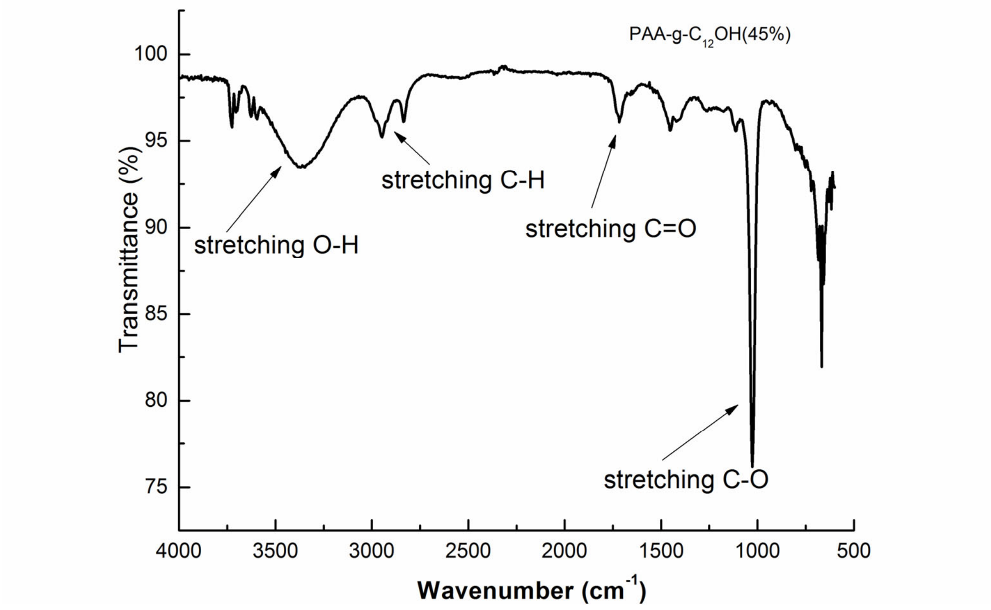

Figure 2S. FT-IR spectrum of the synthesized hydrophobically functionalized poly(acrylic acid) with marked signals attributed to the main bonds.

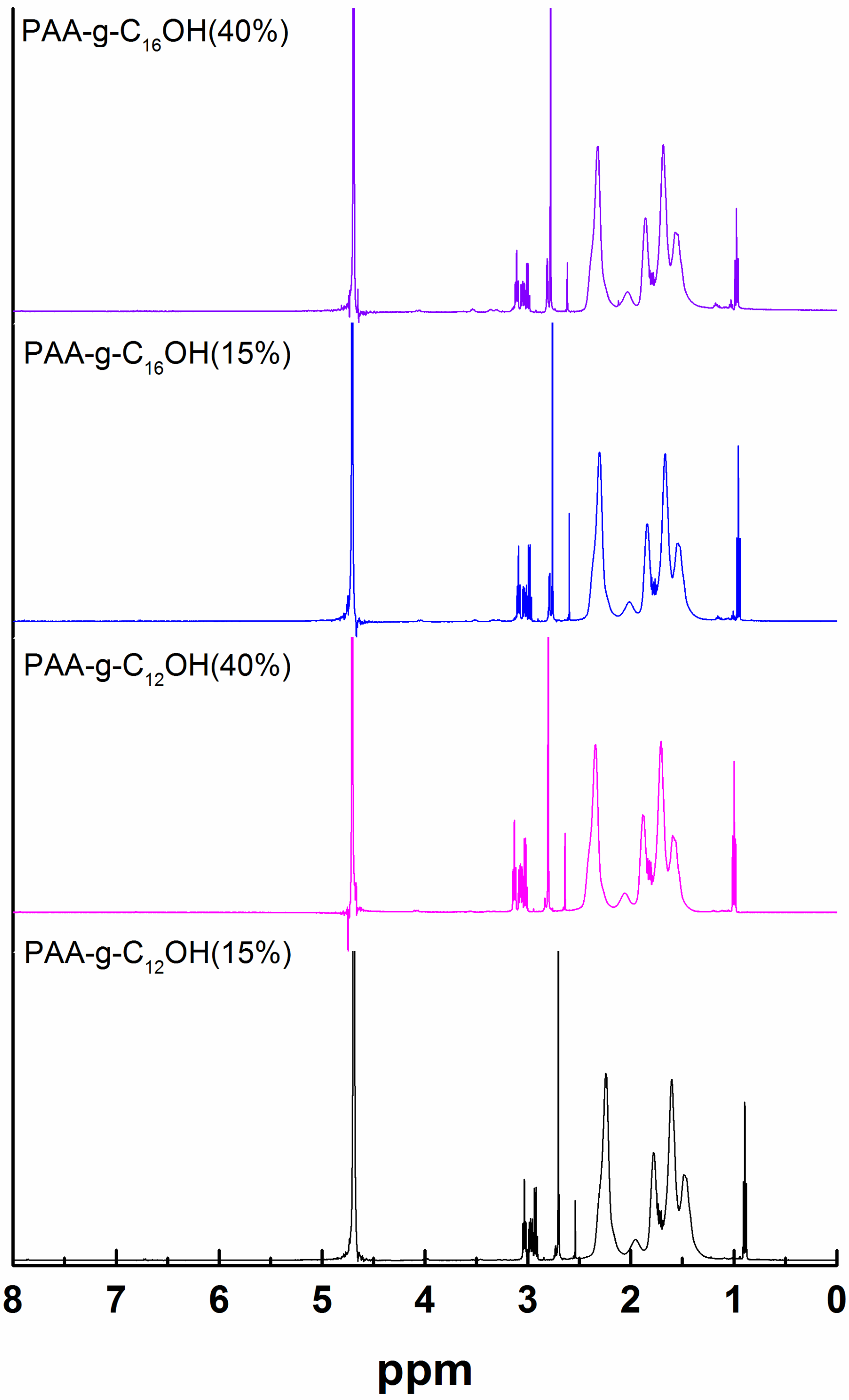

Figure 3S. ^{1}H NMR spectra of the synthesized hydrophobically functionalized poly(acrylic acid) (0 – 8 ppm range)

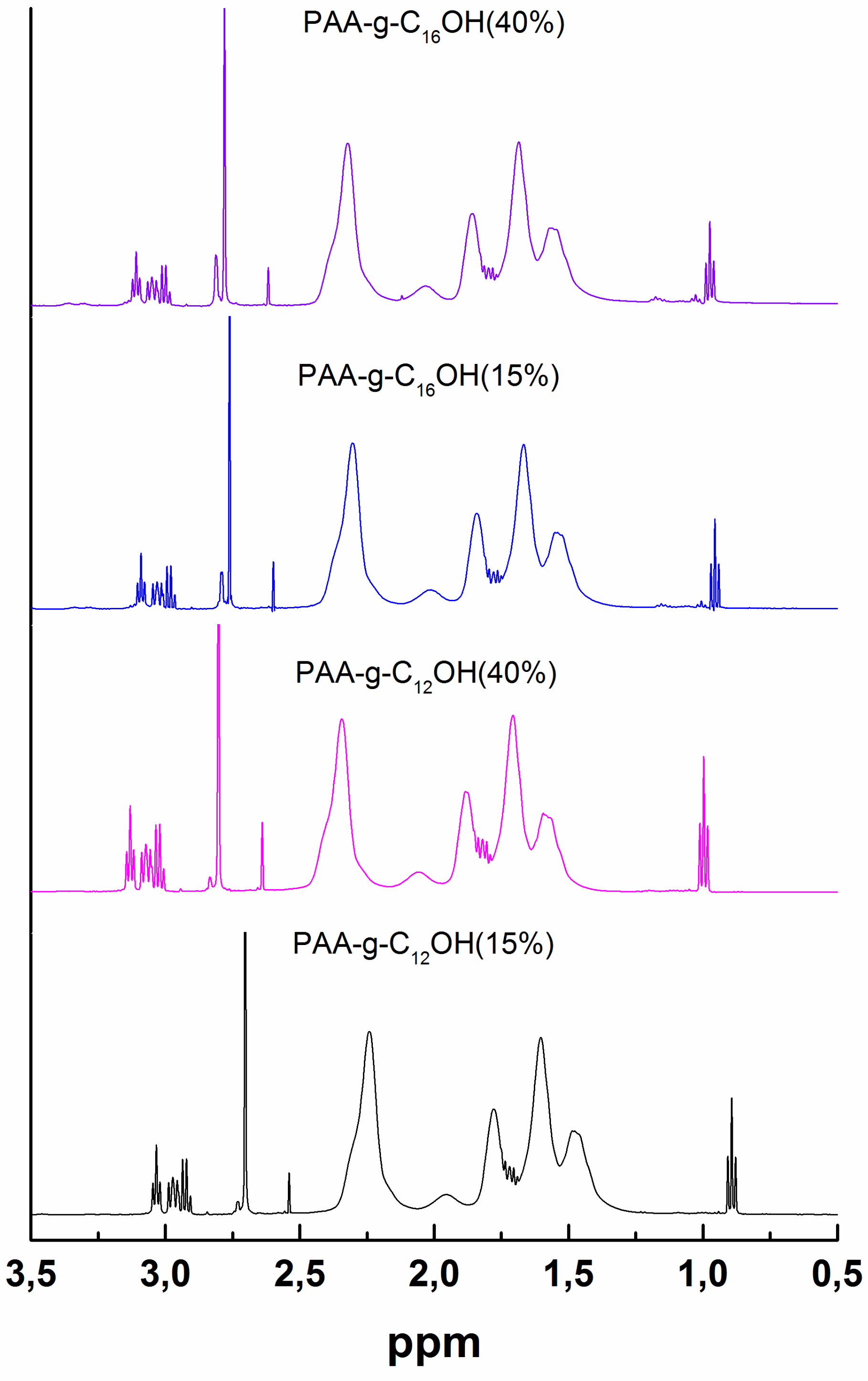

Figure 4S. ^{1}H NMR spectra of the synthesized hydrophobically functionalized poly(acrylic acid) (0.5 – 3.5 ppm range).

2. Determination of diffusion coefficients by fitting to triexponential functions

Table 1S. Determination of diffusion coefficients (D) and hydrodynamic diameters (D_H) by fitting data to appropriate triexponentional models: G^1 (intensity versus linear gradient) or G^2 (intensity versus square gradient).

c [mg/mL]	Model (G^1 or G^2)	PAA-g-C_{12}OH(15%)			PAA-g-C_{16}OH(40%)		
		D [m^2/s]	D_H [nm]	R^2	D [m^2/s]	D_H [nm]	R^2
10	G^1 (3 coefficients)	$5.502*10^{-10}$	0.7	0.99967	$5.368*10^{-10}$	0.7	0.99986
		$5.502*10^{-10}$	0.7		$5.368*10^{-10}$	0.7	
		$5.502*10^{-10}$	0.7		$5.368*10^{-10}$	0.7	
	G^2 (3 coefficients)	$5.498*10^{-10}$	0.7	0.99967	$5.356*10^{-10}$	0.7	0.99986
		$5.498*10^{-10}$	0.7		$5.356*10^{-10}$	0.7	
		$5.498*10^{-10}$	0.7		$5.356*10^{-10}$	0.7	
45	G^1 (3 coefficients)	$4.228*10^{-10}$	1.0	0.99996	$3.814*10^{-10}$	1.0	0.99983
		$4.228*10^{-10}$	1.0		$3.814*10^{-10}$	1.0	
		$6.953*10^{-11}$	5.8		$7.810*10^{-11}$	5.1	
	G^2 (3 coefficients)	$4.230*10^{-10}$	1.0	0.99996	$3.813*10^{-10}$	1.0	0.99983
		$4.229*10^{-10}$	1.0		$3.813*10^{-10}$	1.0	
		$1.248*10^{-11}$	32.1		$7.810*10^{-11}$	5.1	
100	G^1 (3 coefficients)	$4.041*10^{-10}$	1.0	0.99988	$3.229*10^{-10}$	1.2	0.99992
		$2.007*10^{-9}$	0.2		$3.229*10^{-10}$	1.2	
		$3.332*10^{-122}$	$1.2*10^{112}$		$3.229*10^{-10}$	1.2	
	G^2 (3 coefficients)	$3.492*10^{-10}$	1.1	0.99988	$3.236*10^{-10}$	1.2	0.99992
		$1.367*10^{-9}$	0.3		$3.236*10^{-10}$	1.2	
		$-4.327*10^{-10}$	-0.9		$3.236*10^{-10}$	1.2	

3. Molecular modeling studies

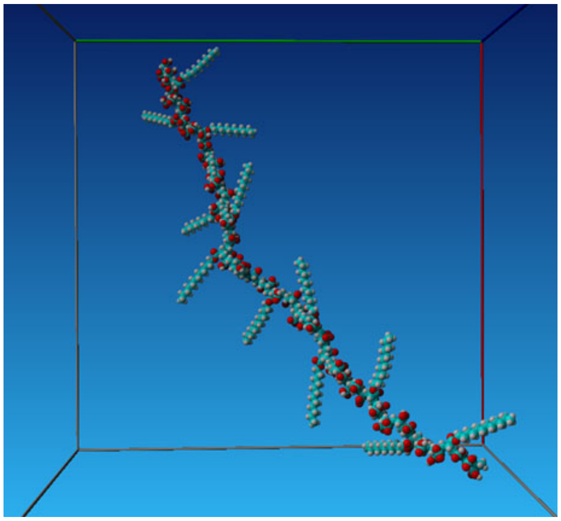

Figure 5S. Initial conformation of hydrophobically modified PAA molecule used for the molecular dynamics simulations.

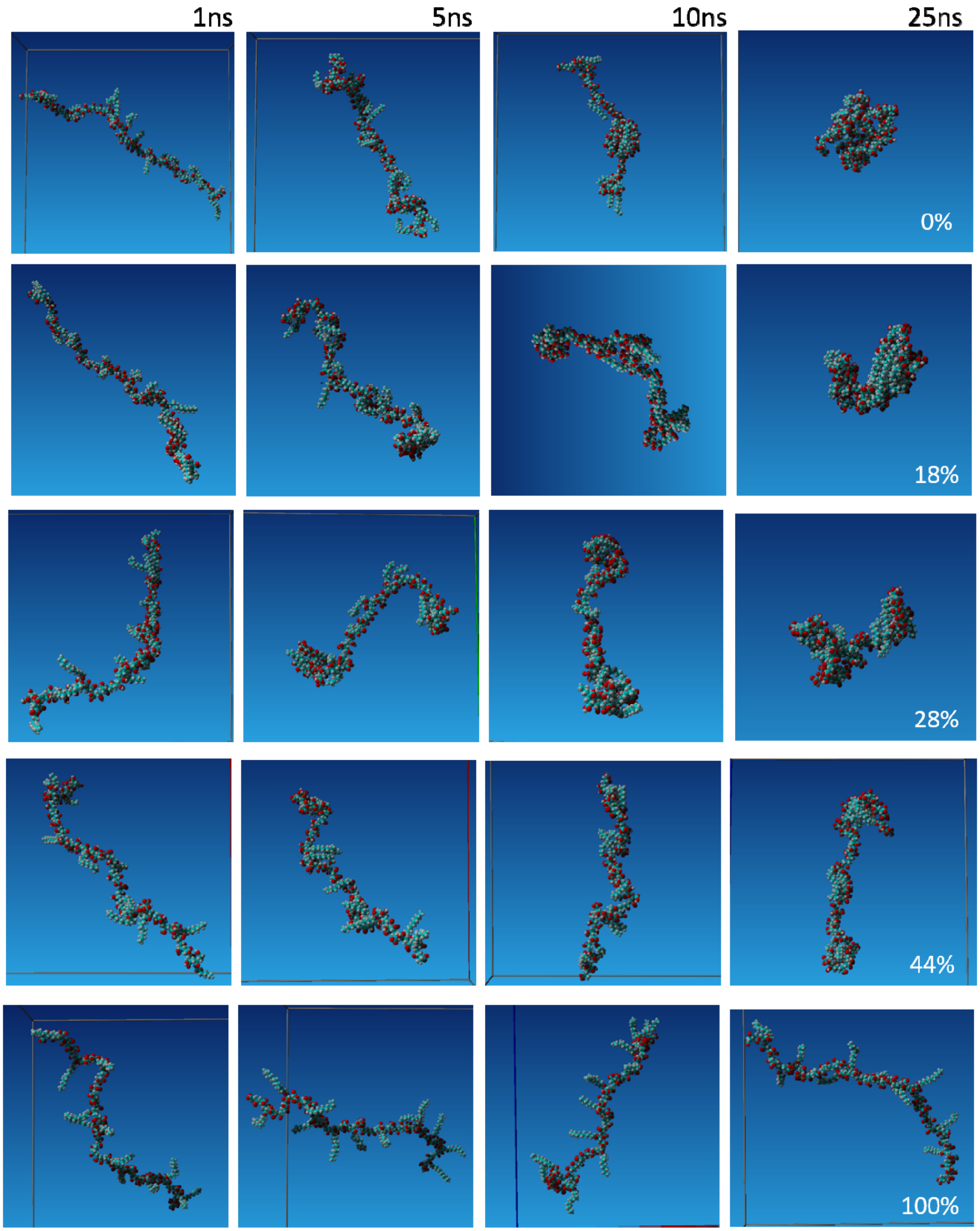

Figure 6S. Snapshots from the molecular dynamics simulations of PAA-g-C_{12}OH(15%) with various degree of charging (marked in the Figure) taken after 1ns, 5 ns, 10 ns and 25 ns of the simulation run.
